# A severity classification model of cervical spondylotic radiculopathy symptoms based on MRI radiomics: A retrospective study

**DOI:** 10.1371/journal.pone.0327756

**Published:** 2025-07-09

**Authors:** Xi Wang, Qiaoli Tao, Huanwen Liu, Huangbo Lin, Honglai Zhang

**Affiliations:** 1 Guangzhou University of Chinese Medicine, School of Medical Information Engineering, Guangzhou, China; 2 Guangzhou University of Chinese Medicine, Intelligent Chinese Medicine Research Institute, Guangzhou, China; Makere University College of Health Sciences, UGANDA

## Abstract

**Objective:**

To develop a severity classification model for symptoms of cervical spondylotic radiculopathy (CSR) based on magnetic resonance imaging (MRI) radiomics and to evaluate the predictive value of MRI radiomics features in the classification of symptoms severity, providing an objective basis for personalized therapeutic interventions.

**Methods:**

This retrospective study included 99 patients diagnosed with CSR, admitted between August 2022 and April 2023. Symptom severity was assessed using the neck disability index (NDI) scale, which facilitated the categorization of participants into mild and severe symptoms groups. A comprehensive set of 3,404 quantitative radiomics features was extracted from four predefined regions of interest (ROIs) using the 3D Slicer software. The least absolute shrinkage and selection operator (LASSO) regression analysis was used to identify the optimal subsets of radiomics features, which were subsequently used to develop a support vector machine (SVM) classification model. Model performance was evaluated using receiver operating characteristic (ROC) curve analysis with area under the curve (AUC) calculations, complemented by accuracy, precision, sensitivity and F1 score evaluations.

**Results:**

Analysis of cervical T2-weighted MRI resulted in the extraction of 3,404 radiomics features from four ROIs. Using the LASSO regression for feature selection, 96 radiomics features were retained for model construction. The most discriminatory characteristics were in the intervertebral discs at levels C4/5, C5/6, and C6/7 in the mid-sagittal plane. The model demonstrated an AUC of 0.91. The accuracy of the model is 0.917, the precision is 0.979, the sensitivity is 0.833, and the F1 score is 0.890.

**Conclusion:**

The severity classification model of CSR symptoms based on MRI radiomics demonstrates robust predictive performance in assessing the severity of CSR symptoms, serving as an effective decision-support tool for guiding personalized therapeutic strategies in clinical practice.

## Introduction

The prevalence of cervical spondylosis has progressively increased, likely influenced by lifestyle changes [1], with cervical spondylotic radiculopathy (CSR) comprising approximately 60%−70% of these cases [2]. Cervical spondylosis manifests itself primarily as neck-shoulder pain, upper limb numbness, and motor dysfunction. In severe cases, it can progress to muscle weakness or atrophy, significantly compromising the quality of life of patients [3,4]. Current evidence indicates that surgical intervention is the most effective strategy to reduce the risk of lifelong disability from cervical spondylosis [5]. Among patients with surgical indications, surgical outcome was better in patients with mild symptoms than in those with severe symptoms [6]. These findings underscore the clinical imperative for a rigorous preoperative assessment of the severity of CSR.

Current assessment methodologies for CSR are mainly based on verbal inquiries and observational assessments by clinicians. However, this subjective methodology, based on patient self-reports and physician-perceived evaluations, inherently limits the objective quantification of the severity of symptoms. Consequently, there has been a concerted scholarly effort to develop objective assessment tools to assess CSR progression.

Magnetic resonance imaging (MRI), due to its noninvasive nature and superior soft tissue resolution, remains the gold standard imaging modality for cervical spine evaluation [7,8]. Previous studies used mainly manually measured imaging signs to investigate the correlations between MRI findings and the severity of CSR symptoms. However, the impact of imaging signs of CSR on patients’ symptoms such as pain and dysfunction has not been established.

Numerous studies have indicated that the imaging findings of CSR may not correlate with the severity of symptoms. For example, certain patients demonstrate clinical manifestations and symptoms of CSR in the absence of detectable abnormalities in imaging modalities [9]. In contrast, other individuals may present with cervical disc herniation with associated nerve compression on imaging but remain asymptomatic. Furthermore, investigations of the association between imaging findings and clinical symptoms frequently yield inconclusive and conflicting results. Yang et al. [10] conducted a longitudinal cohort study involving 223 patients with radiculopathy, reporting that the prevalence rates of Modic changes were 18% at the beginning of the study and 23% at a 1-year follow-up. Their analysis did not uncover any statistically significant association between Modic changes and the severity of neck pain at either time. In contrast, Li et al. [11] compared 266 patients with Modic changes against 338 controls, finding significantly higher rates of neck pain (42.1% vs. 26.6%, P = 0.000) and disc degeneration scores (4.6 ± 2.8 vs. 2.2 ± 2.5, P = 0.032) in the Modic changes group. These contradictory results highlight the ongoing controversy about the relationship between CSR symptoms and imaging features, potentially attributable to subjectivity in physician visual assessments and manual measurements of imaging data, which can introduce bias and limit diagnostic accuracy.

In this study, to overcome the limitations of the above-mentioned approaches, we used a radiomics approach to automatically identify and analyze imaging features. Radiomics, as an emerging field in medical imaging [12], assists clinical diagnosis and decision making by extracting quantitative, high-throughput and ideally reproducible radiological features from medical images. These features are difficult to recognize or quantify through visual inspection alone, thus compensating for the shortcomings of traditional imaging diagnosis based on subjective empirical evaluations [13,14]. The application of radiomics to identify deeper MRI features has proven to be a valuable tool to enhance diagnostic accuracy. Previous studies have shown that radiomics analysis can reveal numerous deep features in MRI images, which may reflect underlying pathophysiological information [15]. In recent years, radiomics has been widely applied to spinal disorders with promising results, including automated classification of degenerative spinal lesions [16–18], differential diagnosis and prognostic prediction of spinal tumors [19,20], diagnostic evaluation of spinal pathologies [21,22], and segmentation of spinal anatomical structures [ 23, 24].

The application of machine learning models has demonstrated excellent performance in the diagnosis and classification of cervical spine diseases.Xie J. et al. [25] proposed a decision support tool based on MRI radiomics for the automatic grading of cervical disc degeneration. Through a retrospective analysis at two centers (435 patients, 2610 intervertebral disc samples), an automatic grading model for cervical disc degeneration based on machine learning was constructed and validated.The random forest algorithm performed best in T2 MRI (AUC 0.91). This demonstrates the value of multimodal MRI radiomics in the grading of cervical degeneration. Hopkins et al. [26] collected cervical spine images from 14 patients with cervical myelopathy and 14 healthy subjects, entering selected imaging features into two neural network models. The results of the model indicate that machine learning-based radiomics offers a promising approach for the diagnosis and prognosis of patients with cervical myelopathy. Alan et al. [27] analyzed the radiomics texture characteristics of 62 patients with cervical spondylotic myelopathy (CSM). The results of the supervised machine learning model demonstrated that the radiomics features exhibited significant correlations with the severity of the preoperative symptoms in patients with CSM, and these radiomics features could serve as imaging biomarkers to assess the severity of the symptoms of CSM. Furthermore, multiple studies have shown that support vector machines (SVMs) are the most effective algorithms to predict treatment response and outcome events in radiomics analysis [28–32].

This study innovatively proposes a machine learning classification model for establishing the severity of CSR symptoms based on MRI radiomics, identifying relevant biomarkers that reflect the severity of symptoms. The method aims to provide a non-invasive early identification, offering an objective reference for preoperative diagnosis, thereby facilitating the development of personalized treatment strategies.

## Materials and methods

### Patients

This retrospective study was approved by the Medical Ethics Committee of the Guangdong Provincial Hospital of Traditional Chinese Medicine (No. ZE2023-043-01). All enrolled patients were informed of the study protocol and provided their written informed consent, as required by the ethical review approval.

**Fig 1:**
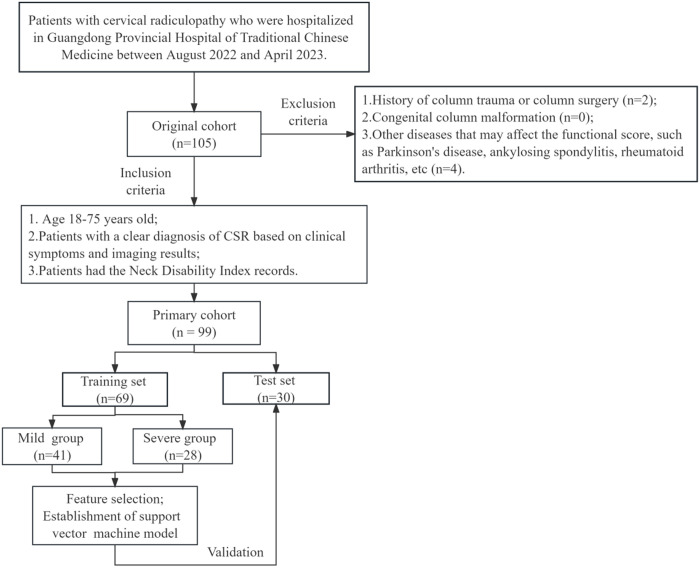
Flow chart of patient selection.

This retrospective study analyzed medical records of 99 cases of CSR at Guangdong Provincial Hospital of Traditional Chinese Medicine, from August 1, 2022, to April 30, 2023. Clinical data included patient age, gender, occupation, and duration of the disease. The imaging data comprised sagittal cervical T2-weighted MRI images of the cervical spine. All included patients met the criteria and exhibited symptoms as described in the Evidence-Based Clinical Guideline for the Diagnosis and Treatment of Lumbar Disc Herniation with Radiculopathy, established by the North American Spine Society (NASS). This study was conducted in accordance with the principles of the Declaration of Helsinki. All data accessed for research purposes were collected between April 1, 2023, and May 31, 2023 (Fig 1).

Inclusion criteria: (1) patients aged 18–75 years; (2) patients diagnosed with CSR based on clinical symptoms and imaging examination results; (3) availability of detailed neck disability index (NDI) score records.

Exclusion criteria: (1) a history of spinal trauma or spinal surgery; (2) congenital spinal deformities; (3) concurrent infections, tuberculosis, tumors, or other systemic diseases; (4) comorbidities that affect functional scores.

### Research methods

#### MRI examination.

All included patients underwent cervical MRI at 3.0 T using Siemens MRI equipment with a head and neck coil. Conventional images of sagittal TSE T1WI, T2WI, and axial T2WI were acquired with the cervical spine in neutral position. The parameters for the sagittal TSE T2WI were as follows: repetition time (TR) of 3500 ms, echo time (TE) of 91 ms, slice thickness of 3 mm, slice spacing of 0.3 mm, 12 slices, field of view (FOV) of 280 × 280 mm, and number of excitations (NEX) of 1. The parameters for the axial T2WI were as follows: repetition time (TR) of 314 ms, echo time (TE) of 17 ms, slice thickness of 2 mm, slice spacing of 0.2 mm, 3 slices, field of view (FOV) of 180 × 180 mm, and number of excitations (NEX) of 2.

#### Symptoms severity information.

In this study, the neck disability index NDI was used to classify the severity of the symptoms among the enrolled patients. NDI is widely used to self-assess disability associated with neck pain. The scale consists of 10 items: pain intensity, personal care, lifting capacity, reading duration, concentration, headache severity, work capacity, driving ability, sleep quality, and participation in recreational activities. The total score ranges from 0 points (indicating no disability) to 50 points (indicating complete disability), with higher scores corresponding to more severe symptoms. It is suitable for various types of cervical spondylosis and has shown good reliability and validity [33]. After completion of the NDI self-assessment, the patients were classified into two groups based on their scores: the mild group (NDI < 30) and the severe group (NDI ≥ 30) [34].

#### Image processing and feature extraction.

**Fig 2:**
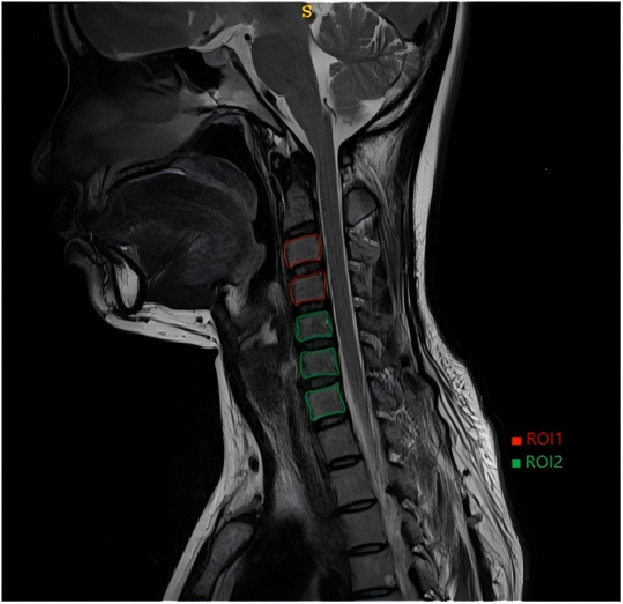
ROI 1 and ROI 2.

**Fig 3:**
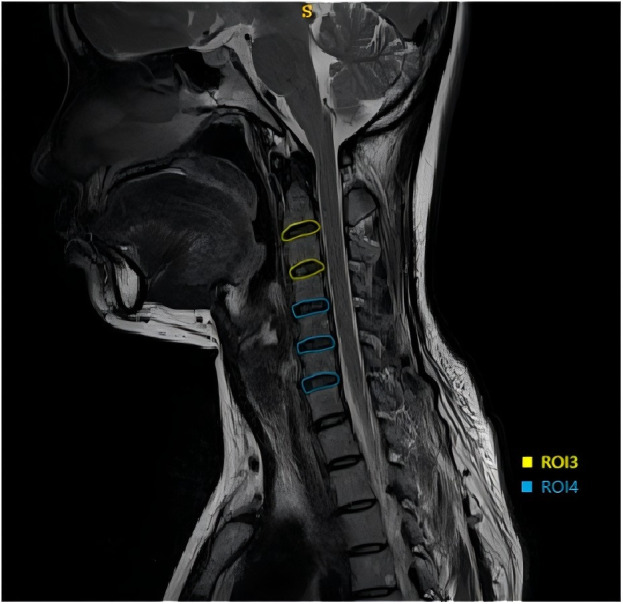
ROI 3 and ROI 4.

The collected images were stored in DICOM format. To minimize the impact of inconsistent pixel values in images from different patients, the MRI images were resampled prior to feature extraction, with a voxel size set to 1 mm × 1 mm × 1 mm. Under the guidance of a radiologist with more than 4 years of experience, 3D Slicer software(https://www.slicer.org/) was used to delineate regions of interest (ROIs) on the T2-weighted images (T2WI) in the sagittal plane and to perform feature extraction. ROIs were defined as follows (Figs 2 and 3):

ROI 1: the third and fourth vertebrae at the mid-sagittal plane.

ROI 2: the fifth, sixth, and seventh vertebrae at the mid-sagittal plane.

ROI 3: the C2/3 and C3/4 intervertebral discs at the mid-sagittal plane.

ROI 4: the C4/5, C5/6, and C6/7 intervertebral discs at the mid-sagittal plane.

The set of features comprised 851 quantitative image parameters per ROI, including 18 first-order features, 14 morphological features, 75 second-order statistical features, and 744 high-order statistical features.

#### Model development and validation.

SVMs are widely used for binary classification tasks in medical data analysis [35]. The algorithm requires optimization of a quadratic programming problem to establish decision boundaries during training [36]. In this study, radiomics features were segmented by maximizing the separation hyperplane in the feature space using SVM, allowing for an accurate classification of symptoms in patients with CSR. The stability of the algorithm on small-sample datasets provided robust technical support for constructing a machine learning-based CSR symptom classification model. The SVM algorithm used the radial basis function (RBF) kernel. Theoretically, models using the RBF kernel can fit any complex data distribution and perform excellently on high-dimensional small-sample data [37]. The radiomics features data in this study conform to the data characteristics suitable for applying the RBF kernel. During the model training process, the training set results are used to observe whether the model experiences over-fitting or under-fitting, ensuring the reliability of the test set results. The regularization parameter C and the RBF kernel parameter γ are tuned to enhance the model performance.

### Statistical analysis

Statistical analysis was conducted on the included patients using SPSS (version 25.0; IBM, Armonk, NY, USA). Differences in demographic data were evaluated using the independent samples t-test or the Mann-Whitney U test, as appropriate. All statistical methods used a significance level of P < 0.05 to determine statistically significant differences.

In radiomics analysis, feature selection is crucial for mitigating overfitting, redundancy, and bias. The extracted features were standardized using StandardScaler to mitigate scale variations between features. The least absolute shrinkage and selection operator (LASSO) algorithm was utilized to select 3,405 radiomics features and construct an optimal subset. The fundamental principle of this method involves applying a penalty function that reduces the coefficients and sets non-contributory variables to zero, thus selecting features that contribute significantly to the target variable from a multitude of candidates [38,39]. In this study, we determined the prediction error of the LASSO regression model with different optimal tuning parameter (λ) using a ten-fold cross-validation method, selected the λ that minimized the error, and the features retained at this value are the selected results of radiomics features. Feature selection and model construction were implemented in Python 3.9 using the scikit-learn and pandas libraries.

## Results

### Baseline characteristics

A total of 99 patients with CSR were enrolled, with a mean age of 47.54 years (42 males, 51.36 ± 9.75 years; 57 females, 49.16 ± 11.58 years). The cohort consisted of 59 cases in the mild group and 40 in the severe group. No significant differences were observed between the groups in terms of gender (P > 0.05) or duration of the disease (P > 0.05). However, the severe group had a higher mean age (53.85 ± 9.99 years) compared to the mild group (47.54 ± 10.73 years; P < 0.01). There was a significant difference in the occupational distribution between the groups (P < 0.05). The baseline characteristics did not show statistical differences between the training and testing set (P > 0.05). (Table 1 and 2)

**Table 1:** Comparison of baseline characteristics between mild group and severe group.

Characteristics	Category	Mild group n = 59(%)	Severe group n = 40(%)	χ^2^/t/z	*P*
**Gender**	Female	31(52.54)	26(65.00)	1.515	0.218
	Male	28(47.46)	14(35.00)		
**Occupation**	Professional technical personnel	9(15.25)	6(15.00)	12.421	0.029
	Office workers	26(44.07)	8(20.00)		
	Business personnel	6(10.17)	1(2.50)		
	Production personnel and manufacturing personnel	3(5.08)	7(17.50)		
	Retirees	8(13.56)	9(22.50)		
	Other personnel	7(11.86)	9(22.50)		
**Age**	/	47.54 ± 10.73	53.85 ± 9.99	−2.950	0.004
**Disease duration**	/	8.000(2.0,36.0)	6.000(1.0,36.0)	−0.353	0.724

**Table 2:** Comparison of characteristics data between training set and test set.

Characteristics	Category	Training set n = 69(%)	Test set n = 30(%)	χ^2^/t/z	*P*
**Gender**	Female	41(59.42)	16(53.33)	0.317	0.573
	Male	28(40.58)	14(46.67)		
**Occupation**	Professional technical personnel	4(13.33)	11(15.94)	6.790	0.237
	Office workers	8(26.67)	26(37.68)		
	Business personnel	1(3.33)	6(8.70)		
	Production personnel and manufacturing personnel	3(10.00)	7(10.14)		
	Retirees	5(16.67)	12(17.39)		
	Other personnel	9(30.00)	7(10.14)		
**Age**	/	49.20 ± 10.83	52.13 ± 10.77	1.239	0.218
**Disease duration**	/	6.000(3.0,42.0)	6.000 (1.0,36.0)	−0.700	0.484

### Radiomics feature selection

**Fig 4:**
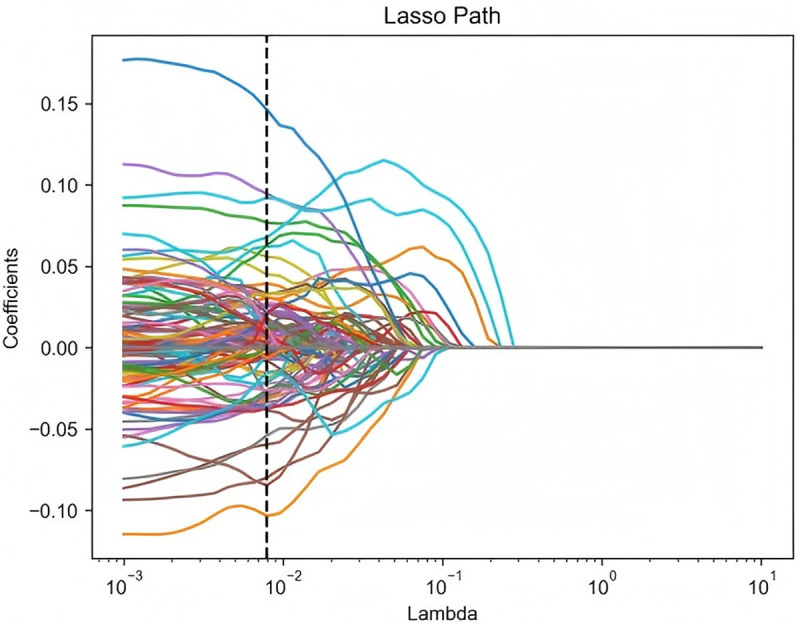
Distribution map of radiomics features least absolute shrinkage and selection operator regression coefficients.

**Fig 5:**
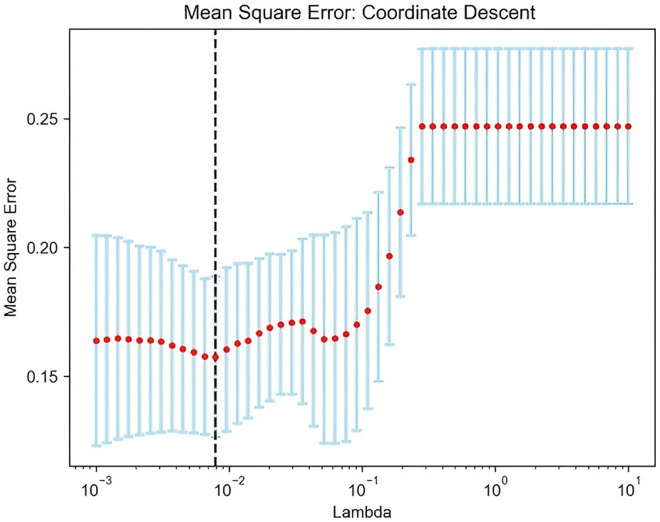
Radiomics feature least absolute shrinkage and selection operator regression cross-validation plot.

**Fig 6:**
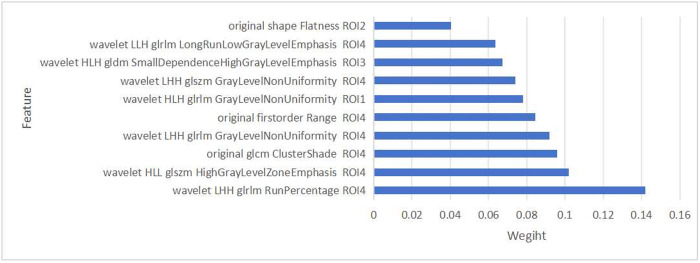
Coefficient diagram of important features (only the top ten features are displayed).

In this study, a LASSO regression analysis was conducted on 3,404 radiomics features extracted from four ROIs, with the optimal tuning parameter (λ) determined via ten-fold cross-validation. The optimal λ value was denoted by a vertical dashed line at 0.0079 (Figs 4 and 5). Subsequently, features with zero regression coefficients were discarded, resulting in the retention of 96 optimal imaging features. The correlation coefficients of the significant features were then ranked based on their importance (Fig 6). The greater the value on the horizontal axis, the higher the contribution rate of the radiomics features.

### Model construction and performance evaluation

**Fig 7:**
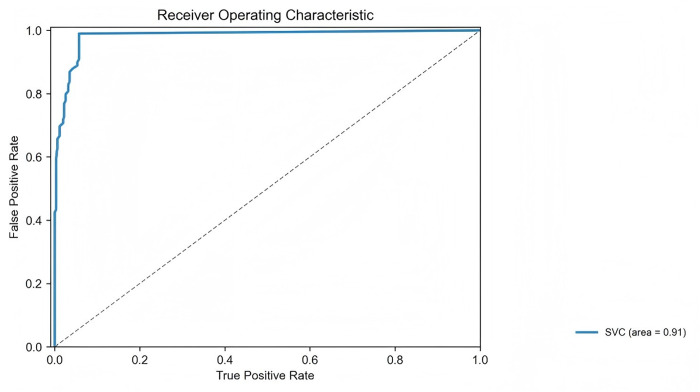
Performance of CSR symptoms severity classification model.

The receiver operating characteristic (ROC) curve of the severity classification model is presented (Fig 7), demonstrating an area under the curve (AUC) of 0.91. The accuracy of the model is 0.917, the precision is 0.979, the sensitivity is 0.833, and the F1 score is 0.890 (Table 3).

**Table 3:** Diagnostic performance of the model in the test set.

Accuracy (95%CI)	Precision (95%CI)	Sensitivity (95%CI)	F1 Score (95%CI)
0.917 (0.895 ~ 0.939)	0.979 (0.962 ~ 0.996)	0.833 (0.790 ~ 0.876)	0.890 (0.861 ~ 0.919)

## Discussion

In this study, we extracted high throughput radiomics features from cervical T2-weighted MRI of patients diagnosed with CSR. We used LASSO regression as a variable selection method to filter these features and constructed an SVM model using selected radiomics features to evaluate its efficacy in predicting the severity classification of symptoms. Given the relatively small sample size, ten-fold cross-validation was employed to minimize overfitting and enhance the model’s generalization capability. The results demonstrated an AUC of 0.91, with an accuracy of 0.917, a sensitivity of 0.833 and an F1 score of 0.890. This indicates that the model is capable of accurately distinguishing the severity of symptoms in patients with CSR, and radiomics features have potential clinical value in the assessment of the severity of symptoms. In clinical practice, the model can predict the disease progression of CSR patients through objective MRI image data, providing a reference for preoperative evaluation. This method not only improves diagnostic efficiency by reducing manual dependency and subjective bias but also promotes the development of personalized treatment plans and offers new insights for scholars to explore the mechanisms of CSR symptom occurrence.

In previous studies, scientists have used radiomics features to classify symptoms of degenerative spinal diseases. For example, Alan N. et al. [27] discovered that radiomics models correlate with preoperative mJOA scores using T2 texture features in CSM patients, indicating their potential as surrogate and objective imaging biomarkers for measuring preoperative functional status. This aligns with our conclusion that MRI-derived radiomics features can serve as biomarkers to assess the severity of symptoms in cervical spondylosis, thus helping clinicians determine patient functional status and allowing timely interventions. Juuso H. J. et al. [29] classified low back pain patients into symptomatic and asymptomatic groups using a logistic regression classifier based on T2 MRI textural features, achieving an accuracy of 83%. On the contrary, the relationship between imaging signs and severity of symptoms in CSR remains complex, with no definitive conclusions established [30]. In terms of model selection, SVM models outperform logistic regression by capturing complex non-linear relationships between predictor variables and outcomes [40]. SVMs achieve this through the non-linear mapping of samples into high-dimensional spaces and the identification of optimal hyperplanes for class separation [41]. This method uniquely addresses challenges in high-dimensional modeling with limited samples, exhibiting low error rates and robust generalization capabilities.

Regarding the selection of ROIs, we categorized them into four regions based on cervical segments, including vertebral bodies and intervertebral discs. An et al. [42] evaluated vertebral endplate classification (defined by changes in the intensity of MRI signal) in 283 patients with cervical spondylosis, demonstrating a significant correlation between cervical endplate signal alterations and neck pain (OR 5.356; P < 0.001), with predominant changes observed in the C5-C7 segments. This biomechanical phenomenon probably arises because the C5-C7 endplates carry greater axial loads than the superior segments, as their vertebrae exhibits less restricted mobility, transmitting thus greater mechanical forces [43]. This observation led us to hypothesize that the middle and lower cervical segments may contain more radiomics features associated with the severity of symptoms.

The analysis of baseline clinical data revealed a significant age difference between the mild and severe groups (P < 0.05), confirming previous evidence that age is a risk factor for the progression of cervical spondylosis [44].

The factors that determine pain and dysfunction in CSR are not well understood but are often thought to involve interactions between various complex elements, such as mechanical compressive stimuli, chemical inflammatory mediators, and psychological states [45–47]. Our research indicates that multiple imaging markers have predictive value for different levels of severity of CSR symptoms. In particular, the radiomics features extracted from ROI 4 (intervertebral discs C4/5, C5/6 and C6/7) had the highest proportion of significant features, aligning with our initial hypothesis. This suggests that the middle and lower cervical discs contain more biological information related to symptoms, which could potentially improve the classification efficiency of the models.

Cervical intervertebral discs pathology has been widely recognized as a crucial contributor to neck pain [48,49]. J.S. Lawrence et al. [50] conducted a study in the UK of 3,375 patients with cervical spondylosis, establishing significant correlations between cervical disc degeneration and axial pain. Liu et al. [51] performed a retrospective analysis of the morphological parameters of T2-weighted MRI in 88 patients with CSR, revealing correlations of the postoperative symptoms score with the orientation of the central axis of herniation and the central angles formed in the spinal cord. In contrast, Makarand V. Risbud et al. [46]. proposed a chemical-inflammatory mechanism, concluding that degenerate discs secrete pro-inflammatory cytokines (TNF, IL-1α, IL-1β, IL-6, and IL-17), thus amplifying intradiscal inflammatory responses and subsequent development of radicular pain. Collectively, these studies support the crucial role of cervical disc alterations in spondylosis-related symptomatology.

Despite the promising results of this study, several limitations should be acknowledged. First, the exclusive reliance on single-center data collection introduces potential selection bias, necessitating future multicenter studies to enhance sample diversity and model generalizability. Second, while manual ROI segmentation was performed by professionally trained researchers under the supervision of a radiologist, this approach remains susceptible to variability between observers. Further research can employ inter-observer or intra-observer consistency analysis to filter out features with subjective biases in the segmentation of regions of interest. Machine learning techniques can also be applied for automated segmentation. For example, Xie J. et al. [25] applied a refined MedSAM model to automatically segment intervertebral discs with an average Dice coefficient of 0.93, offering fresh perspectives to reduce the subjective influence in experiments.

Additionally, multimodal approaches can improve model performance. Xie J. et al. [25] proposed a decision support tool based on MRI radiomics for the automatic grading of cervical disc degeneration. The model using the random forest algorithm performed best on T2 MRI (AUC 0.91) and the combined model incorporating multimodal features further improved performance, achieving an AUC of 0.95 and an accuracy of 89.51% in the test set. This suggests that in radiomics studies, integrating multimodal features can help uncover more biomarkers that affect disease diagnosis, thereby enhancing model performance. Concurrently, the existing methodology concentrates exclusively on radiomics of the sagittal plane through ROI-based analysis, overlooking the evaluations of the volume of interest (VOI) of cervical structures. This two-dimensional approach may miss critical three-dimensional radiomics features related to spatial anatomical relationships. To address this issue, we propose incorporating multisequence multimodal imaging with VOI-based radiomics, allowing the extraction of depth-resolved features and the development of optimized classification models for the prediction of the severity of CSR symptoms.

## Conclusion

In this study, we developed a severity classification model of CSR symptoms based on MRI radiomics. The model demonstrated strong predictive performance for the severity levels of CSR symptoms, serving as a valuable decision support tool to guide the formulation of personalized therapeutic strategies in clinical practice.

## Supporting information

S1 Data Research data. (XLSX)
